# Protein Nanomechanics

**DOI:** 10.3390/nano12193524

**Published:** 2022-10-08

**Authors:** Gabriel Žoldák

**Affiliations:** Center for Interdisciplinary Biosciences, Technology and Innovation Park, Pavol Jozef Šafárik University, Trieda SNP 1, 040 11 Košice, Slovakia; gabriel.zoldaks@upjs.sk

For a comprehensive understanding of protein function and dynamics, it is crucial to study their mechanical properties [1,2,3,4,5,6,7,8,9,10,11]. The exploration of internal protein nanomechanics is challenging for several reasons. First, proteins are tiny, nanometer-sized objects, so the expected forces acting within protein substructures are in the piconewton range. To study such small forces, unique physical experimental methods able to operate at the nanoscale have to be developed, such as atomic force microscopy and nanosurgical manipulations [12], as well as optical and magnetic tweezers, to name a few. To mechanically interrogate proteins, in addition to specialized physical methods, site-specific bioconjugation assays are needed (reviewed in [13]). Site-specific bioconjugation guarantees a precise definition of the pulling direction, which is needed due to the strong mechanical anisotropy of proteins [14,15,16]. The mechanical anisotropy of proteins is tremendously important in easing the unfolding activity of essential AAA+ proteases in a cell [17]. Experimental data obtained from single-molecule force spectroscopy contain enormous amounts of unique information, which is otherwise highly difficult to obtain using any other methods [18]. When a protein folds and unfolds at different force ranges, an underlying 1D free-energy landscape can be recovered after careful analysis due to Hammond–Leffler transition state movements [19]. In addition to studies regarding functional folded proteins, the mechanical properties of peptides on water/solid interfaces can be quantified using force spectroscopy [20].

Recent progress regarding theoretical AI-based approaches for the prediction of 3D structures [21] has created further questions about the prediction of protein dynamics and the stability of internal substructures. In this Special Issue, we show that it is possible to infer the internal dynamics of mechanical units from a 3D structure using normal mode analysis (NMA), and this theoretical approach can be used to explore the nanomechanical properties of proteins [22]. Moreover, using several machine learning models, a successful prediction of mechanically stable folding units was demonstrated by only utilizing the information in the primary sequence of a protein [23].

Van der Sleen and Tych [13] reviewed available bioconjugation assays for the preparation of protein-DNA tethers used in single-molecule force methods. To study a protein under load, the protein is often attached to a manipulable probe such as the tip of an AFM cantilever or micron-sized bead, controlled by laser optical trapping or a magnetic field. As significant interactions between a solid surface and a protein can exist, the molecular construct is often designed to implement a flexible linker between the protein and the AFM tip or the trapped bead. For optical tweezers, the flexible linker also protects the protein from being too close to the highly focused laser beam, which prevents denaturation and photo-damage of the protein. The authors describe how molecular linkers are utilized in single-molecule force spectroscopy. Based on their nature, linkers can be divided into two types: non-covalent or covalent linkers (aka handles). The major difference between these handle types is mechanical stability. Non-covalent molecular handles display rupture forces around hundreds of piconewtons, while covalent handles have roughly ten times higher mechanical stability of around several thousand piconewtons. Handles have to secure a temporally stable physical connection between the protein and probe, making it possible to exert and control mechanical forces. The review summarizes and describes different types of non-covalent and covalent handles and discusses the pros and cons of individual bioconjugation strategies. It also provides practical considerations and rationale for the choice of bioconjugation assays for the efficient preparation of protein-DNA tethers.

Gala and Žoldák [23] applied supervised and unsupervised machine learning approaches to distinguish between residues in mechanical stable and unstable fragments of Hsp70 based on sequence information alone. The mechanical properties of fragments were studied experimentally in previous work. For successful learning, the local context of amino acids has to be included in the training phase. In a very simplified version of local context, the authors used a moving average window and provided new sets of features. Three supervised machine learning methods were used: random forest, support vector machine and logistic regression (LR). The LR model showed the best accuracy and was subjected to cross-validation, confirming the model’s excellent performance. The further application and development of machine learning models based on primary sequences alone may help identify stable substructures in proteins and design artificial stable mini proteins.

Single-molecule force experiments can explore protein folding and unfolding under mechanical force. However, such experiments are challenging, cost-demanding and require many years of highly specialized expertise. Bauer and Žoldák [22] describe an option to examine protein mechanics through a theoretical approach called normal mode analysis supplemented by buried volume analysis. The paper compares theoretically obtained results with experimental outcomes. Three previously studied non-homologous proteins were examined using the NMA: T4 lysozyme (T4L), Hsp70 and the glucocorticoid receptor domain (GCR). The NMA results for T4L and Hsp70 were compared with steered MD simulations conducted previously, and the authors found very good agreement with the main results obtained using the simulations and experiments. NMA identifies substructures that correlate with experimentally identified unfolding intermediates for the GCR. In summary, NMA is a promising, computationally cheap method that can be used to examine the mechanics of protein structures and can support the structural analysis of the results from mechanical studies regarding proteins.

Atomic force spectroscopy was used to examine the mechanical properties of two peptides on the surfaces of gold nanoclusters (Au NCs) as small as 2 nm [20]. Such small gold nanoclusters (Au NCs) are interesting due to their optical and electronic properties. For the mechanical study, the following peptides were used: a long and flexible elastin-like polypeptide (ELP)20, consisting of a repeat unit of Val-Pro-Gly-Xaa-Gly derived from human tropoelastin, and a rigid peptide (EAAAK)3 with a length of 7.5 nm. The peptides differ in their elastic properties; the Young’s modulus of (ELP)20-Au NCs is about 50 MPa, which is higher than that of (EAAAK)3-Au NCs (ca. 35 MPa). The authors successfully conjugated a long and flexible (ELP)20 on an ultra-small gold nanocluster via one-pot synthesis. Potentially suitable peptides can be used as linkers for protein immobilization, which may allow for further characterization via single-molecule force spectroscopy.

Non-equilibrium pulling data and derived force-dependent kinetic rates measurements show a systematic discrepancy between the total distance between the native (N) and the unfolded state (U) from elastic models and the sum of the measured distances for folding and unfolding kinetics. Rico-Pasto et al. [19] performed single-molecule force spectroscopy for highly kinetically stable protein barnase to explain the observed discrepancy. The authors observed that the transition state (TS) shifts with force relative to the unfolded state, which provides a plausible explanation for the discrepancy. The movement of the TS position obeys the Leffler–Hammond postulation, which says that two adjacent states move closer to each other along the reaction coordinate as the energy difference between them becomes smaller [24]. Hence, the distance between the native state and TS decreases at higher forces.

Sziklai et al. [12] applied high-resolution atomic force microscopy and nanosurgical manipulation to examine the structure and mechanics of the M-complex and associated titin proteins. The authors concluded that the M-complex is a stable structure that corresponds to the transverse unit of the M-band organized around the myosin thick filament. Specific parts of titin that originate from the M-complex can extend and unfold their domains. In addition, based on their pulling experiments, they suggest that the M-complex may be viewed as a compact supramolecular reservoir of extensible filaments and hence can be involved in diverse mechanical functions within the muscle sarcomere. In addition, the development of nanosurgical manipulation methods can support the deep exploration of the structure and mechanics of complex molecular assemblies.

Microtubule disassembly and protein degradation are essential processes in the cell, which are mediated by highly specialized hexameric nanomachines. Varikoti et al. [17] conducted computational studies of two AAA+ nanomachines: microtubule-severing protein spastin and the caseinolytic protease ClpY. The results of their molecular simulations at the atomistic and coarse-grained scales show that both proteases accomplish the unfolding of their substrates by taking advantage of mechanical anisotropy. In the case of spastin, optimal severing action is achieved through the specific orientation of the machine versus the substrate. In the ClpY-mediated unfolding of the substrate dihydrofolate reductase (DHFR), the force is applied along the soft mechanical direction after the disruption of mechanically strong β-sheet interfaces.

To summarize, as shown by the research articles in this Special Issue, protein nanomechanics is a fruitful emerging concept which describes the internal dynamics of highly mechanically anisotropic proteins. The further development of experimental methods and bioconjugation strategies will strongly contribute to developing our fundamental understanding of complex protein nanomachines and protein behavior at solid interfaces. Based on our knowledge of protein mechanics, we should be able to design and develop nanomachines and proteinaceous surfaces with tailored-made functional and elastic properties.
